# Next Steps for Access to Safe, Secure DNA Synthesis

**DOI:** 10.3389/fbioe.2019.00086

**Published:** 2019-04-24

**Authors:** James Diggans, Emily Leproust

**Affiliations:** Twist Bioscience Corporation, San Francisco, CA, United States

**Keywords:** biosecurity, synthetic biology, DNA, cyberbiosecurity, policy

## Abstract

The DNA synthesis industry has, since the invention of gene-length synthesis, worked proactively to ensure synthesis is carried out securely and safely. Informed by guidance from the U.S. government, several of these companies have collaborated over the last decade to produce a set of best practices for customer and sequence screening prior to manufacture. Taken together, these practices ensure that synthetic DNA is used to advance research that is designed and intended for public benefit. With increasing scale in the industry and expanding capability in the synthetic biology toolset, it is worth revisiting current practices to evaluate additional measures to ensure the continued safety and wide availability of DNA synthesis. Here we encourage specific steps, in part derived from successes in the cybersecurity community, that can ensure synthesis screening systems stay well ahead of emerging challenges, to continue to enable responsible research advances. Gene synthesis companies, science and technology funders, policymakers, and the scientific community as a whole have a shared duty to continue to minimize risk and maximize the safety and security of DNA synthesis to further power world-changing developments in advanced biological manufacturing, agriculture, drug development, healthcare, and energy.

## Introduction

In 2010, the United States Department of Health and Human Services (HHS) published the Screening Framework Guidance for Providers of Synthetic Double-Stranded DNA (U. S. Department of Health Human Services, 2010). The Guidance provided a set of recommended practices to companies synthesizing double-stranded DNA to encourage such companies to screen both their customers and requested sequences. Several of the largest DNA synthesis companies came together to form the International Gene Synthesis Consortium (IGSC), a trade industry organization intended to promote the beneficial application of gene synthesis technology while safeguarding biosecurity.

The IGSC published the Harmonized Screening Protocol (International Gene Synthesis Consortium, 2009) to provide additional tactical detail around the implementation of Guidance-compliant customer and sequence screening. The Protocol specifies that synthetic gene sequence orders will be screened against the IGSC's Regulated Pathogen Database (RPD), a data set assembled and maintained by the IGSC of sequences and organisms subject to regulatory control or licensing. The Protocol further specifies that IGSC companies will only supply genes from regulated pathogens to “*bona fide* government laboratories, universities, non-profit research institutions, or industrial laboratories demonstrably engaged in legitimate research.” Since its initial publication, the Protocol has been updated only once (International Gene Synthesis Consortium., 2017) to (among other minor edits) add language affirming that IGSC member companies agree not to synthesize any sequence with “best match” to variola, the virus that causes smallpox, as the disease was declared eradicated by the WHO in 1980. In addition to the Protocol, the IGSC has also developed an extensive onboarding process for potential new members to assist companies and institutions as they build new screening systems.

In the years since the publication of the Guidance, both the DNA synthesis industry and the larger synthetic biology community have rapidly advanced in terms of capability and scale. These advances create new opportunities to revolutionize many industries—from healthcare to industrial chemicals and even digital data storage. With new capabilities come new challenges to the recommendations originally spelled out in the Guidance. As the trajectory of technological advancement will inevitably continue to steepen, here we visit potential options for next steps to advance and continue to secure the manufacture of synthetic DNA and prevent the risk of misuse.

Twist Bioscience (a member company and officer of the IGSC) has witnessed first-hand how challenging some of the Guidance recommendations can become at increasing scale. Those difficulties must be surmounted while maintaining customer and sequence screening accuracy and still achieving the tight delivery timelines demanded by fierce competition within the global DNA synthesis industry.

As scale drives down cost per base pair, the relatively fixed cost of screening plays a more direct role in overall price. These costs are driven by both customer and sequence screening—commercially-available customer screening solutions still require a great deal of manual review of false positive findings. These false positives create a floor on the possible reduction in labor cost of new customer onboarding. Current sequence screening algorithms are computationally expensive and, given the high false positive rate, the results of sequence screening can be complicated to interpret. These generally require a PhD in bioinformatics both for implementation as well as day to day interpretation of hits. This makes scaling interpretation, in the absence of high-quality sequence annotation, a very expensive proposition.

Evolving technologies have blurred the lines between the gene- and oligo-length synthesis products originally addressed in the Guidance. These include ever-simpler methods for the assembly of pools of oligo-length DNA into gene-length DNA and the use of truly massive oligo pools for data storage. The data storage use case, in particular, will drive a substantial global increase in the number of unique oligo sequences under manufacture, making it ever easier to acquire the oligo-length sequences necessary to assemble genes that would otherwise be subject to regulatory control.

## Evolving Industry Best Practices

We believe continued forward-thinking improvements in the biosecurity safety net provided by DNA synthesis order screening will require participation from all interested parties: synthesis companies themselves, policy makers, science and technology funders (both public and private), and the broader synthetic biology community.

### Gene-Length Sequence Screening Performance

The Guidance and the IGSC have together accomplished a great deal in harmonizing the screening practices of the largest synthesis companies. The current IGSC onboarding protocol for new members even includes a set of test sequences to ensure that prospective member institutions have built their custom sequence screening systems with a solid level of accuracy. It is challenging, however, to determine when a custom-built screening system is “good enough”—especially given that the details of each screening implementation remain private to the implementing company. In addition, the recommendations in the Guidance do not specify particular performance metrics in terms of overall sensitivity and specificity or the degree to which sequence alteration or the source of annotation should impact screening results.

This is not the fault of the Guidance—it is extremely difficult to express in the abstract a set of performance characteristics for a system intended to screen the universe of all possible sequences. The cybersecurity and defense communities, facing similar challenges of performance estimation for complex systems, have turned to *red teaming* as a way of answering whether a given system is sufficient to accomplish a protective goal (Zhang and Gronvall, 2018). The best way to estimate whether a skilled adversary can bypass a system is to ask skilled individuals to attempt to do just that. Previous recommendations (Koblentz, 2017) have explicitly called for IGSC companies to regularly test procedures or submit to third-party audits; we believe regular red teaming by a sophisticated third party is an effective means to address these concerns. Twist has recently engaged in an extensive red teaming of our sequence screening system (publication in review) and shared the results with other IGSC members to help further improve our respective systems. We strongly recommend that synthesis companies engage in periodic red teaming as a means of assessing evolving risk of vulnerabilities in screening systems.

Red teaming has additional secondary value: sequences shown to bypass a screening system then serve as effective regression tests during follow-on software development once vulnerabilities have been patched. Regression testing is a software testing paradigm (Yoo and Harman, 2012) designed to ensure that future changes to software systems do not create new ways for previously discovered vulnerabilities to be exploited. Building and scaling a modern sequence screening system is a complex undertaking and requires using distributed computing and third-party annotation resources, both of which increase the risk of regressions during software development and maintenance. Consistent regression testing along with a suite of edge-case test sequences can help manage this risk.

### Screening Oligo-Length Sequences

The 2010 Guidance set a lower bound of 200 nucleotides on the length of sequence with “best match” to organisms appearing on any of the various regulatory control lists. This was intended to strike a balance between ensuring safe manufacture of gene-length sequences while also avoiding the burden of screening for manufacturers of shorter DNA sequences. In the intervening years, however, capacity for generating enormous, diverse pools of oligo-length sequences has grown (Organick et al., 2017) while lower-cost methods for assembling high-quality, gene-length sequences from oligo pools have been developed and matured (Plesa et al., 2018). Together, these two factors create a potential vulnerability: what would be considered controlled for gene-length synthesis under current regulatory and technical systems would be permitted for synthesis as an oligo pool and could be converted into a gene length sequence by assembly in a modestly equipped molecular biology laboratory.

Proposals for screening shorter DNA sequences have been accompanied in the past by a fear of high false positive rates. This would be true were individual oligos subject to screening one at a time—we propose instead that collections of individual oligo orders and oligo pools first be subject to computational *de novo* assembly (i.e., *in silico* assembly). Such techniques (Bonham-Carter et al., 2014; Nimmy and Kamal, 2015) from the Next Generation Sequencing (NGS) community allow for computationally efficient answers to the question of actual interest: *what could I assemble (in vitro) out of this pool of short sequences*? The output from *de novo* assembly methods are longer “contig” (i.e., contiguous) sequences. These contigs should then be subjected to standard gene-length sequence screening; any red flag alert for a contig should trigger customer follow-up identical to that in the Guidance for gene-length sequences.

## Research Funding Priorities

Research funding by governments and other institutions can play a powerful role in making customer and sequence screening easier to build or acquire and more efficient (and therefore less costly to operate) while increasing the accuracy of risk estimation.

### Predicting Risk in Context

The Guidance and all current sequence screening implementations focus on determining whether a given sequence is a “best match” to an entry on a list of organisms subject to regulatory control. These lists include the U.S. Federal Select Agent Program (FSAP) and the Australia Group treaty for harmonized export control. Such lists of organisms, in the context of sequence screening, are generally proxies for a broader goal: determining whether a given ordered sequence could be used to cause significant harm.

For a regulatory control regime to focus on this much more salient challenge, we must move beyond lists of known pathogens and instead focus on the biological context and known “routes to harm.” These can be as simple a single protein (e.g., in the case of ricin) or as complex as the potentially hundreds of genes required for a bacterial pathogen (e.g., the genes required by *Francisella tularensis* to cause tularemia). This annotation requires a committed, ongoing effort to catalog, in detail, the ways in which proteins and genetic networks can be used to cause harm in contexts subject to regulatory control. The knowledge of these mechanisms and the genes they require is highly specialized and diffuse across academic, government, and industrial experts. We understand the assembly of this knowledge in a single, shared location to be both incredibly important and incredibly challenging.

Sustained funding and commitment will be required to build and maintain a database of risk-associated sequences, their known mechanisms of pathogenicity and the biological contexts in which these mechanisms can cause harm. This database (or at a minimum a screening capability making use of this database), to have maximum impact on global DNA synthesis screening, must be available to both domestic and international providers. Arguments have previously been made that such a collection would make misuse of biology easier for bad actors. Modern deep learning methods, while powerfully predictive, often require enormous amounts of high-quality, curated training and specialized statistical expertise to make accurate predictions on complex outcomes. Allowing access only to synthesis companies or others with a “need to know” establishes a threshold for who can work on these challenges and limits the degree of global creativity that can be applied to the challenge of predicting biological outcomes from collections of primary sequence. We believe the value provided by the collection and public dissemination of this information, in terms of empowering machine learning and other risk estimation efforts, far outweighs any increased potential for attempted misuse.

We have excellent examples of this approach in the cybersecurity community: Common Vulnerabilities and Exposures (CVE) (MITRE, 1999) and the National Vulnerability Database (NVD) (National Institute of Standards and Technology, 2000). CVE and NVD publicly catalog known vulnerabilities and code exploiting those vulnerabilities. These data are used to build ever-more-capable intrusion detection systems and to inform software development practices to avoid creation of new vulnerabilities. We believe this same paradigm would work well in a biological context.

As this database grows, additional investment in statistical methods for risk estimation will result in approaches with increasing accuracy in predicting harm. These systems should move from predicting risk on primary DNA sequences to include predicting possible harmful outcomes from genetic circuit designs or even from engineered microbial communities. The Intelligence Advanced Research Projects Agency, IARPA, is funding early work in this area via its Functional Genomic and Computational Assessment of Threat (FunGCAT) program (IARPA, 2016). We strongly encourage funding of complementary and follow-on approaches.

The metaphorical similarity to the cybersecurity domain is not, admittedly, perfect. Patching software vulnerabilities is far easier and less expensive than “patching” biological vulnerabilities via vaccines or novel medical countermeasures. This does not mean, however, that simply enumerating the genes required for a particular “route to harm” is sufficient information to enable bad actors—a flat list of genes involved in a pathogenic outcome is not a recipe. Furthermore, there are large scale efforts underway including the DARPA Pandemic Prevention Platform (P3) program (DARPA., 2017) to enable just this sort of rapid response to novel pathogens. We maintain that the upside of providing this level of detail—low-cost, uniformly accurate, peer-reviewed sequence screening—more than offsets any potential for additional information hazard.

### Sharing Risk Estimation Across Companies

IGSC companies have long recognized the risk of “venue shopping”—that a bad actor intent on acquiring dangerous sequences could submit an order to multiple companies in the hope of finding a company whose screening system will permit the order. The IGSC addresses some of this risk by having each company alert the other IGSC companies to any order causing significant concern.

This still leaves a potential vulnerability in terms of an individual ordering sub-threshold sequences from multiple companies and then carrying out final assembly themselves. The only way to gain a shared awareness of this kind of activity would be to devise a system for sharing assembly and alignment data across companies. Such a system, however, would need to be hosted by a trusted third party and not disclose business-sensitive information including the underlying sequences themselves, the total volume of sequence from any individual company, or any decision-making to manufacture on the part of contributing companies.

Technical solutions to this problem could include sharing only sub-sequences (referred to as “k-mers” in bioinformatics, i.e., sub-sequences of length k) as well as more exotic mathematical methods including homomorphic encryption. Homomorphic methods (that is, methods allowing for computation on data that remains encrypted throughout) would theoretically allow for alignment of sequences to a set of controlled references without disclosing the exact composition of the query sequence (Esvelt, 2018; Titus et al., 2018). In the absence of actual homomorphic alignment methods and given recent work in pseudo-alignment for RNA-Seq data (Bray et al., 2016), we believe pseudo-alignment approaches show the most near-term promise. They operate on k-mers (rather than requiring full sequences) and scale efficiently by, paradoxically, *not* focusing on determining detailed homology-based matches of a query sequence to a database of possible origin sequences. Instead, they estimate only the likelihood that a given sequence came from a given origin sequence—the statistical “best match.” This aligns precisely with the challenge posed to synthesis sequence screening.

### Democratizing Access to Sequence Screening

Maximizing the security of global DNA synthesis will require an ever-larger tent as new synthesis companies are created and grow around the world to serve local or other niche markets. Building a screening system, however, can be expensive and non-trivial. Especially for companies whose business model focuses on thin margins or low volume, the current economics (even with extensive IGSC advice and support) strongly dis-incentivize screening. To lower this barrier to entry for screening, we must solve two problems: software for carrying out the screen and access to high-quality, up-to-date annotation on controlled toxins, viruses, and bacteria. The previous recommendation for ongoing commitment and public availability of a database of “routes to harm” satisfies the second of these criteria. The first could be satisfied by the creation of a small but competitive market for software-as-a-service-based solutions or even open source software allowing a company to quickly install and screen (at low volume) with high accuracy.

Open source would also allow peer-review of algorithmic approaches to screening, further insuring against the risk of software vulnerabilities driving unintended access to sequences. Open software development, however, would require access to curated screening data both to be used by the tool operationally as well as to rigorously test the implementations to ensure they cannot be subverted via clever construct design. This need for validation could create communities of individuals attempting to build sequences that might expose vulnerabilities—this, again, leverages a useful pattern in the cybersecurity world of “bug bounty” programs meant to encourage the constructive application of creativity to identify and report software weaknesses.

## International Norms and the Security Mindset

Long-standing efforts within the synthetic biology community raising awareness of the potential security applications of these technologies has paid dividends and should be expanded. The community must ensure that DNA synthesis companies are not seen as the only stopgap to misuse. Companies designing genetic circuits and novel organisms often are, and should continue to be, active participants in security-related threat evaluation and estimation of potential misuse of the technologies they invent, mature, and sell. We recommend that the focus of the 2010 Guidance on “know your customer” should apply more broadly and explicitly to the entire synthetic biology industry and supply chain.

In addition to building this awareness within companies, it is crucial to continue and expand education efforts on the importance of biosecurity and development of a security mindset in synthetic biology. The International Genetically Engineered Machine (iGEM) competition Safety and Security and Human Practices efforts have educated thousands of young scientists on the importance these kinds of security considerations in synthetic biology. The Engineering Biology Research Council (EBRC) in the United States recently held a Department of Homeland Security (DHS)-funded workshop focused on further improving consideration of security in synthetic biology, recommending that graduate-level scientific education should explicitly teach security awareness to young researchers.

The workshop also highlighted the potential value of asking in grant applications that, in addition to considering the *safety* implications of proposed work (i.e., how might this work accidentally harm yourself or others), applicants also demonstrate that they have thought through the *security* implications (i.e., how might this work be used to *intentionally* harm others). This can improve early awareness of broader security implications of new technologies and foster community discussion and interaction on the risk and benefit trade off and evaluation by a broader community of ethicists or other relevant experts.

Internationally, the Nuclear Threat Initiative has recently launched their Global Biosecurity Innovation and Risk Reduction Initiative intended to “develop, publicize and promote concrete and normative actions to reduce global catastrophic biological risks associated with advancements in technology” (Nuclear Threat Initiative., 2018). Such large-scale, well-funded international activities are extremely valuable in establishing and harmonizing expectations of security considerations and behavioral norms across national borders.

## Conclusion

With increasing scale and complexity in manufacture of synthetic DNA, and in synthetic biology more broadly, comes a responsibility to ensure these technologies continue to be used responsibly. Here, we have outlined a multi-faceted approach to advance the technology, policy, educational, and social environments that help guard against potential misuse. We recommend periodic red teaming to ensure an understanding of the current performance characteristics of DNA sequence screening systems. Additional science and technology investment can build the annotation resources and algorithms necessary to continue to improve both the accuracy and affordability of screening. By lowering the cost of screening and making open source annotation resources and tools available, a much wider array of synthesis companies will be able to screen their orders.

We also recommend that the U.S. government extend guidance to include screening of oligonucleotide pools. This approach should emphasize hypothesis generation via *de novo* assembly from one or many oligo pools rather than focusing alerting on single, short sequences (which can lead to high false positive rates). We further suggest that the U.S. government guidance to “know your customer” apply broadly across the synthetic biology supply chain. In addition, we actively encourage efforts to teach and promote the evaluation of the security implications of new synthetic biology techniques or materials as part and parcel of being a practicing synthetic biologist.

Together, these steps will ensure screening and security practices scale both in terms of the rapidly growing number of global synthesis requests as well as evolving with increasing human knowledge of biological systems and functional components. This multifaceted approach will better serve our shared duty to use synthetic DNA to protect and improve the well-being of people and our planet.

## Author Contributions

All authors listed have made a substantial, direct and intellectual contribution to the work, and approved it for publication.

### Conflict of Interest Statement

JD and EL are employed by Twist Bioscience. Twist Bioscience is a board member of the International Gene Synthesis Consortium (IGSC). The views expressed here are not necessarily those of the IGSC.

## References

[B1] Bonham-Carter O. Steele J. Bastola D. (2014). Alignment-free genetic sequence comparisons: a review of recent approaches by word analysis. Brief. Bioinformatics 15, 890–905. 10.1093/bib/bbt052 23904502 PMC4296134

[B2] Bray N. L. Pimentel H. Melsted P. Pachter L. (2016). Near-optimal probabilistic RNA-seq quantification. Nat. Biotechnol. 34, 525–527. 10.1038/nbt.3519 27043002

[B3] DARPA (2017). Pandemic Prevention Platform (P3). Available online at: https://www.darpa.mil/program/pandemic-prevention-platform

[B4] Esvelt K. M. (2018). Inoculating science against potential pandemics and information hazards. PLoS Pathogens 14:e1007286. 10.1371/journal.ppat.1007286 30286188 PMC6171951

[B5] IARPA (2016). Functional Genomic and Computational Assessment of Threats (Fun GCAT). Available online at: https://www.iarpa.gov/index.php/research-programs/fun-gcat

[B6] International Gene Synthesis Consortium (2009). Harmonized Screening Protocol. Available online at: https://portal.sgidna.com/files/IGSC%20Harmonized%20Screening%20Protocol.pdf

[B7] International Gene Synthesis Consortium (2017). Harmonized Screening Protocol V2. Available online at: https://genesynthesisconsortium.org/wp-content/uploads/IGSCHarmonizedProtocol11-21-17.pdf

[B8] Koblentz G. D. (2017). The *de novo* synthesis of horsepox virus: implications for biosecurity and recommendations for preventing the reemergence of smallpox. Health Security 15:6. 10.1089/hs.2017.0061 28836863

[B9] MITRE (1999). CVE - Common Vulnerabilities and Exposures (CVE). Available online at: https://cve.mitre.org/. (accessed January 7, 2019)

[B10] National Institute of Standards Technology (2000). National Vulnerability Database. Available online at: https://nvd.nist.gov/. (accessed January 7, 2019)

[B11] Nimmy S. F. Kamal M. S. (2015). Next generation sequencing under *de novo* genome assembly. Int. J. Biomath. 08:1530001 10.1142/S1793524515300018

[B12] Nuclear Threat Initiative (2018). NTI Launches New Global Biosecurity Innovation and Risk Reduction Initiative. Available online at: https://www.nti.org/newsroom/news/nti-launches-new-global-biosecurity-innovation-and-risk-reduction-initiative/. (accessed October 30, 2018).

[B13] Organick L. Ang S. D. Chen Y.-J. Lopez R. Yekhanin S. Makarychev K. (2017). Scaling up DNA data storage and random access retrieval. BioRxiv:114553. 10.1101/114553

[B14] Plesa C. Sidore A. M. Lubock N. B. Di Zhang, Kosuri, S. (2018). Multiplexed gene synthesis in emulsions for exploring protein functional landscapes. Science 359, 343–347. 10.1126/science.aao5167 29301959 PMC6261299

[B15] Titus A. J. Flower A. Hagerty P. Gamble P. Lewis C. Stavish T.. (2018). SIG-DB: leveraging homomorphic encryption to securely interrogate privately held genomic databases. PLoS Comput. Biol. 14:e1006454. 10.1371/journal.pcbi1006454 30180163 PMC6138421

[B16] U. S. Department of Health Human Services (2010). Screening Framework Guidance for Providers of Synthetic Double-Stranded DNA. Available online at: https://www.phe.gov/Preparedness/legal/guidance/syndna/Pages/default.aspx

[B17] Yoo S. Harman M. (2012). Regression testing minimization, selection and prioritization: a survey. Softw. Test. Verification Reliabil. 22, 67–120. 10.1002/stvr.430

[B18] Zhang L. Gronvall G. K. (2018). Red teaming the biological sciences for deliberate threats. Terrorism Political Violence 1–20. 10.1080/09546553.2018.1457527

